# Reduction in lignin content and increase in the antioxidant capacity of corn and sugarcane silages treated with an enzymatic complex produced by white rot fungus

**DOI:** 10.1371/journal.pone.0229141

**Published:** 2020-02-21

**Authors:** Erica Machado, Paula Toshimi Matumoto Pintro, Luis Carlos Vinhas Ítavo, Bruna Calvo Agustinho, João Luiz Pratti Daniel, Nadine Woruby Santos, Janaina Macieiro Bragatto, Matheus Gonçalves Ribeiro, Lúcia Maria Zeoula

**Affiliations:** 1 Department of Animal Science, State University of Maringa, Maringa, Paraná, Brazil; 2 Faculty of Veterinary Medicine and Animal Science, Federal University of Mato Grosso do Sul, Ipiranga Village, Campo Grande, Mato Grosso do Sul, Brazil; University of Illinois, UNITED STATES

## Abstract

The objective was to evaluate the effect of the addition of 0, 10, 20, and 30 mg.kg^-1^ of natural matter of a lignocellulosic enzymatic complex produced by the white rot fungus on the chemical composition, cumulative gas production in vitro, and antioxidant compounds of corn and sugarcane silages. After being chopped and treated with the enzymatic complex, the plants were packed in vacuum-sealed bags. After 60 days, the mini silos were opened and the samples were dried in a forced ventilation oven at 55 °C for analysis of the proposed parameters. The experiment was conducted in a completely randomized design with four replicates per treatment. In the corn silage, there was a linear reduction in the lignin concentration. In the sugarcane silage showed a reduction of 12% in the lignin concentration, a linear reduction in the hemicellulose content, and a decrease of 8% in the cellulose concentration compared to the control treatment. The lignin monomers had linear increases in the syringyl:guaiacil ratio. This reflected on significant increases in the concentration of the non-fibrous carbohydrates and the A + B1 fraction of the carbohydrates, and a reduction in the C fraction. The in vitro gas production increased, the time of colonization and initiation of in vitro fermentation linearly decreased in both silages. The phenolic compounds and the antioxidant capacity increased linearly with the addition of the enzymes in both silages. The addition of the lignocellulolytic enzymes to the silages caused changes in the cell wall, resulting in improvements in the in vitro fermentative parameters, besides the additional effect on the antioxidant capacity. There was an effect of the addition of the enzymes on the evaluated fodder, and the best concentration was, on average, 20 mg kg^-1^ MN for corn silage and 10 mg kg^-1^ NM for sugarcane silage.

## Introduction

The use of the fibrous fraction of feed by ruminants is related to the ability of rumen microorganisms to synthesize and secrete enzymes that hydrolyze the cell walls of plants. However, in diets having a high fiber and dry matter content, the use of feed is inefficient [1]. One of the solutions to try to solve part of this problem is the use of exogenous fibrolytic enzymes in the feed of ruminants, especially in ensiled materials. This alternative has been proposed as a strategy to improve the availability of the substrate and to contribute to the increase in the lactic fermentation of silage, as well as to increase its nutritive value [2, 3].

The enzymatic additives can contain a single group of enzymes, a combination of various groups of enzymes (hemicellulases, cellulases, amylases, pectinases, and proteases), or combinations of enzymes and a bacterial inoculant for use in the ensiling process. Most of the enzymes used as additives come from a microbial (*Bacillus* ssp) or fungal (*Trichoderma* sp and *Aspergillus* sp) byproduct or an extract that has one or several types of enzymatic activity [4].

However, the enzymatic complexes with cell wall action that are currently marketed are composed primarily of cellulases and hemicellulases and do not contain enzymes that act on lignin. Lignin represents a physical barrier against the entry of microorganisms due to its close relationship with cellulose and hemicellulose [5], and might limit the digestion of polysaccharides.

Thus, in addition to improving the nutritional quality of the feed, the breakdown of lignin involves the cleavage of the covalent bonds of lignin, resulting in the formation of units with low molecular weight and phenolics that have great potential to improve the quality of the food, increasing its antioxidant properties [6].

Thus, the hypothesis of the present study is that the presence of enzymes that degrade lignin in enzymatic lignocellulolytic complexes, in addition to increasing the energetic value of forage silage used in ruminant feed, can also add antioxidant effects to these feeds. The objective of this study was to evaluate the effect of the addition of increasing concentrations (0, 10, 20, and 30 mg kg^-1^ fresh matter—FM) of a lignocellulosic enzymatic complex produced by the white rot fungus on the chemical composition, cumulative gas production in vitro, and antioxidant compounds in corn and sugarcane silages.

## Materials and methods

The experimental protocols developed in this research fully complied with the ethical principles of animal experimentation, elaborated by the Brazilian College of Animal Experimentation–COBEA and were referred to the Ethics Committee on Animal Use in Experimentation, State University of Maringa, for consideration under the number approval 009/2013.

The experiment was conducted at the Department of Animal Science of the State University of Maringá (UEM, Maringá, PR, Brazil). Field activities took place in the Bovine Nutrition Sector and Meso-Regional Center for Excellence in Milk Technology of the Experimental Farm of Iguatemi and in the Laboratory of Feed and Animal Nutrition Analysis. The in vitro fermentative parameters were analyzed at the Federal University of Mato Grosso do Sul (UFMS, Campo Grande, MS, Brazil).

The lignocellulolytic enzyme complex used in this experiment was developed by our research group, using the fungus mycelium (*Pleurotus ostreatus* (Jacq.) P. Kumm), purchased from Brasmicel, located in Suzano—SP, Brazil and maintained at 4°C in potato-dextrose-agar (PDA) medium. The fungus was incubated with a carbon source (coast cross grass hay—*Cynodon Dactylon* (L.) Pers) and KIRK medium (KH_2_PO_4_, 2g.L^-1^; MgSO_4_ * 7H_2_0, 0.5g.L^-1^; CaCl_2_, 0.1g.L^-1^; Dibasic ammonium tartrate 0.5g.L^-1^; yeast extract 0.2g.L^-1^; pH 5.6–5.8) at 28°C for 12 days for enzyme production (Table 1).

**Table 1:** Composition of the enzymatic complex (U/min/g of enzymatic complex) produced by the white rot fungus used in the silages.

Enzyme	U/min/g of enzymatic complex
Laccase	191.3
Manganese peroxidase	15.4
Lignin peroxidase	12.6
carboxymethylcellulase	206.2
Mannanase	111.1
Xylanase	202.0

Enzyme production occurred by incubating the fungus with a carbon source (*Cynodon Dactylon*—coast cross grass- hay) and KIRK medium at 28°C for 12 days under aerobic conditions.

The enzymatic complex was used after drying in a lyophilizer and, as excipient, cornstarch or sugar was used for addition to corn silage and sugarcane, respectively. Thus, all treatments received the addition of 15 cm³ of enzyme complex (powder). For the calculation of the concentrations added to the forages (0, 10, 20 and 30 enzymatic activity (min). Kg^-1^ fresh matter—FM), was considered the sum of the activities of the 6 enzymes (lacase, manganese peroxidase, lignin peroxidase, carboxymethylcellulase, mannanase and xylanase), evaluated individually.

The determination of laccase activity was performed by spectrophotometric analysis after oxidation of 2,2′-azino-bis (3-ethylthiazoline-6-sulfonate) (ABTS), where were used 1.6 mL of McIlvaine Buffer pH 4.0 (room temperature) with 0.2 mL of ABTS solution (20mM) and 0.2 mL of crude enzyme solution, and the reading was performed 420 nm wavelength [7], after 5 minutes reaction. Manganese peroxidase activity was determined by the Wariishi method [8], where were added 0.6 mL of sodium malonate buffer (50mM) pH 4.5 (room temperature) with 1.2 mL of crude enzyme solution, 0.60 mL of MnSO4 (4.5 mM) and 0.30H_2_O_2_ (9mM); the reaction occurred for 5 minutes and the reading was performed at 270 nm. Lignin peroxidase (LiP) activity was determined by the modified Tien method [9] through the oxidation of veratrylic alcohol to veratrilaldehyde (3,4 dimethoxybenzaldehyde), where 0.75 mL of sodium tartrate buffer (10 mM) pH 3.0 with 0.5 mL of crude enzyme solution, 0.25 mL of veratrylic alcohol (3 mM) and 0.10 mL H_2_O_2_ (5 mM) were added, Absorbance was measured after 5 minutes of reaction at 310nm. For the determination of cellulose, the procedure recommended by Ghose [10] was used, using as substrate the carboxymethylcellulose where 0.250 mL of crude enzyme solution with 0.250 mL carboxymethylcellulose (0.5% in 50mM sodium acetate buffer pH 5.8) was added the reaction was conducted for 30 minutes, and the amount released glucose was measured by reaction with dinitrosalicylic acid, according to Miller [11]; The standard curve was performed with pure glucose (2, 4, 6, 8, 10, 12, 14, 16, 18 and 20 μmol / mL). The enzyme activity of xylanase was determined according to Damiano [12], where a reaction mixture containing 0.1 mL crude enzyme solution and 0.9 mL Birchwood-Sigma xylan (1% in 50 mM sodium acetate buffer, pH 5.4) was incubated for 30 minutes at 50 ° C. The released reducing sugar was quantified by the DNS method [11], the standard curve was constructed with xylose solution (2, 4, 6, 8, 10, 12, 14, 16, 18 and 20 μmol / mL). The enzymatic activity of mannanase was determined according to Rättö [13], where a reaction mixture containing 0.1 mL crude enzyme solution and 0.9 mL galactoglucomannan (0.5% in 50 mM sodium acetate buffer, pH 5.4) was incubated for 30 minutes at 50 ° C. The released reducing sugar was quantified by the DNS method [11], the standard curve was constructed with mannose solution (1.38; 2.77; 4.16; 5, 55, 6.94, 8.33, 11.11 and 13.38 μmol / mL.

The experiment was conducted in a completely randomized design with four replicates per treatment. The statistical analysis of the studied variables were performed by a regression using the PROC MIXED of SAS (Statistical Analysis System, version 9.0.) to evaluate the linear and quadratic responses to the increase in enzymatic complex levels in the silages.

The corn (whole plant) was harvested with 310 g kg^-1^ dry matter (DM) and sugarcane (CTC 25 variety) was harvested by manual cutting with an 18.4 Bx; both were chopped in a stationary forage harvester to cut the forage into pieces approximately 2 cm in length. The sugarcane was treated with a bacterial inoculant containing *Lactobacillus buchneri* in order to reduce alcoholic fermentation.

Both forages were weighed (approximately 500 g) and treated with the lignocellulolytic enzyme complex, which was in contact with the material to be ensiled, about 20 to 30 minutes before vacuum application. The samples were ensiled in vacuum sealed bags (TecMaq, TM-250, São Paulo, Brazil) to ensure the absence of oxygen inside the mini pouches. The silage was stored in the mini silos for 60 days, and the samples were later collected for further chemical analysis. Samples of the material before ensiling and of the silages were dried in a oven at 55 °C for 72 hours and ground in a Wiley-type knife mill so that the particle size was approximately 1 mm. Afterwards, their chemical composition was determined. The dry matter (DM) content of the samples was determined in a forced ventilation oven according to the procedure 934.01 [14]. The ash content was determined by combustion at 600 °C for 6 hours according to the method of AOAC 924.05 [11]. The determination of total N followed the AOAC procedure 990.03 [14]. The concentrations of neutral detergent fiber (NDF) were measured using thermostable amylase, without sodium sulfite [15]. The concentration of acid detergent fiber (ADF), without correction for the ash content, was determined according to procedure 973.18 of the AOAC [14]. The ether extract (EE) content was determined according to method 7.060 of the AOAC [14]. To determine the insoluble nitrogen in the ADF, the ADF residue was used for the determination of total nitrogen, according to AOAC [14].

In order to obtain a more accurate result, the analyses to determine the lignin concentration were performed by two different techniques: potassium permanganate soluble lignin (PPSL) and acetyl bromide soluble lignin (ABSL). PPSL was determined using residue from the determination of the ADF, which was in contact with a solution of potassium permanganate, according to the methodology described by Van-Soest and Wine [16]. The quantification of ABSL followed the technique described by Fukushima and Kerley [17] and the absorbance reading was performed in a spectrophotometer (PC 300 ThermoScientific, Waltham, MA, USA) at 280 nm. The lignin concentration was determined according to a standard curve and expressed in mg cell wall lignin g^-1^.

The monomeric composition of lignin was determined by oxidation with nitrobenzene according to Bubna [18], all the samples were filtered through a 0.45 μm filter and analyzed by High performance liquid chromatography (HPLC) (Agilent 1100 Series, Massy, France), using column ACE C18-AR, 150 mm x 4.6 mm x 5μm, Aberdeen, Scotland. The mobile phase used was 4% methanol/acetic acid in water (20/80, v/v), with a flow of 1.2 mL min^-1^ for 20 min for isocratic analysis. Quantifications of *p*-hydroxybenzaldehyde (H), isovaniline (I), guaiacyl (G), and syringaldehyde (S) were performed at 290 nm using the corresponding standards. The results were expressed as mg of the cell wall monomer g^-1^. From these results, the S/G ratio was calculated.

The hemicellulose fraction was calculated by the difference between the NDF and ADF, and the cellulose fraction was calculated by the difference between the ADF and lignin. The total carbohydrate (TC) content was calculated using the following equation: *TC* = 100 − (% *CP* + % *EE* + % *ash*), [19]. The non-fibrous carbohydrates (NFC) were determined by the difference between the TC and NDF, and the fractionation of carbohydrates was performed according to System CNCPS, in which:

TC=OM−(EE+CP);FractionA+B1=100−(C+B2);

$$\begin{array}{l} Fraction\;B2=100\;x\;(\left(NDF\;\left(\%\;DM\right)\right)\;–\;NIND\;\left(\%\;CP\right)\;\times \;0.01\;\times \;CP\;\left(\%\;DM\right)\;\\ -\;NDF\;\left(\%\;DM\right)\;\times \;0.01\;\times \;LIGNIN\;-\;ABSL\left(\%\;NDF\right)\;\left(\%\;DM\right) \end{array}$$

FractionC=(100×NDF(%DM)×0.01×LIGNIN−ABSL(%NDF)×iNDF/TC(%DM))

To quantify the indigestible NDF (iNDF) used in the calculation of carbohydrate fractionation, the samples were incubated *in situ* in two cattle for 288 h. After incubation, the samples were placed in an Ankom^®^ automatic fiber analyzer (A200) along with the addition of neutral detergent for the quantification of NDF [15].

The carbohydrate recovery was estimated by adapting the equation proposed by [20]:

RCWF=(CWop×DMop)×100(CWcl×DMcl)

Where: RCWF = recovery index of the cell wall fraction, CWop = fraction of the cell wall in the opening, DMop = content of dry matter at the opening, CWcl = fraction of the cell wall at closing, and DMcl = content of dry matter at closing.

For the analysis of the antioxidant compounds, the bioactive molecules of the samples were extracted using 40% acetone for 4 h on a tube shaker and the extract was then centrifuged at 1200g for 10 min. Such extraction conditions were previously determined in a pilot study aiming to obtain the best results for most of the antioxidant variables in the samples of this study.

Quantification of the total polyphenols was performed by the colorimetric method described by Singleton and Rossi [21]. The results obtained are expressed in mg GAE (gallic acid equivalent).

The quantification of flavonoids was performed according to Woisky and Salatino [22], modified by Sanchéz et al. [23]. The results obtained are expressed in mg of QE (quercetin equivalent).

The reducing power of the samples was determined according to Zhu et al. [24], with modifications by Santos et al. [25], and Gallic acid was used as the standard.

The antioxidant capacity using the ABTS reagent was determined as described by Rufino et al. [26]. The results are expressed as Trolox^®^ equivalent.

For the analysis of cumulative *in vitro* gas production, three ruminal cannulated cattle were used as donors of ruminal liquid. Two field replicates (two batteries) were carried out in the apparatus, with rumen liquid from cattle kept on *Brachiaria brizantha* pastures with corn silage supplementation and concentrate based on corn and soybean meal and minerals at will. Each run was weighed and used 0.5 g of sample in triplicate, incubated with McDougall’s [27] buffer solution (artificial saliva100 mL in each vial) and ruminal fluid (25 mL in each vial). Before closing the modules of the apparatus, CO_2_ was gassed to better simulate the ruminal environment. The digestion kinetics were evaluated over the 48 h of incubation, through the cumulative *in vitro* gas production in the fermentative process of each feed by means of a wireless computer system, equipped with a pressure transducer and radio frequency communication (ANKOM^®^ RF, Gas production system). The pressure data, in psi, were collected every 5 min and converted into mL gas/100 mg incubated dry matter. The fermentation parameters corresponding to the different fractions analyzed were obtained according to a bicompartmental logistic model proposed by Schofield et al. [28]:

CGPTotal=[A/(1+exp(2+4*B*(Lag−time))]+[D/(1+exp(2+4*E*(Lag−time)))]

Where: total = cumulative gas production in vitro (mL/100 mg DM incubated), A = gas volume of the fast degradation fractions (soluble sugars and starch), B = rate of degradation per hour of the fast fraction, Lag = time of colonization and beginning of fermentation in hours; D = gas volume of slow degradation fractions (cellulose and hemicellulose); and E = rate of degradation per hour of the slow fraction;

## Results

The results of the carbohydrate recovery of corn and sugarcane silages, after the fermentation process, are presented in Table 2. The recovery of the corn cell wall (NDF) decreased linearly, as shown in the regression equation. For the sugarcane silage, the neutral detergent fiber recovery showed a 14% decrease compared to the control treatment. These results were mainly due to a decrease in lignin.

**Table 2:** Recovery of the fractions (%) of carbohydrates of the silages treated with lignocellulolytic enzymatic complex.

Parameters	Enzimatic activity (U. min). kg^-1^ NM				SEM	P	
	0	10	20	30		Linear	Quadratic
Corn Silage							
Neutral detergent fiber	74.1	69.4	60.8	56.2	7.78	<0.01	0.11
Total Carbohydrates	93.8	93.9	91.1	89.5	4.40	0.02	0.20
Non-fibrous carbohydrates	99.8	106	110	116	8.40	<0.01	0.10
Cellulose	94.9	85.9	82.7	83.2	2.24	0.21	0.02
Hemicellulose	54.7	51.5	48.4	45.3	8.86	0.01	0.16
Lignin (ABSL)	84.1	66.4	54.0	54.3	1.65	0.02	0.07
Sugarcane Silage							
Neutral detergent fiber	77.7	68.7	67.4	69.6	14.9	0.08	0.02
Total Carbohydrates	77.2	74.5	72.1	74.0	3.62	0.12	0.01
Non-fibrous carbohydrates	82.4	83.6	90.8	94.4	6.66	0.01	0.24
Cellulose	85.8	77.5	78.1	80.1	5.62	0.14	0.02
Hemicellulose	63.9	47.2	42.2	47.3	6.36	0.21	0.01
Lignin (ABSL)	81.2	69.5	66.9	69.0	2.84	0.21	0.03

Lignin (ABSL): lignin determined by reaction with acetyl bromide; SEM: standard error of mean.

In corn silage, the addition of the lignocellulosic enzymatic complex (at levels from 0 to 30 mg kg^-1^ NM) caused linear and quadratic positive effects on the chemical composition of the silage (Table 3), especially on the fractions of cell wall (P<0.05). There was a 22% decrease in the acid detergent insoluble nitrogen (ADIN) content, which presented a quadratic behavior. The levels of NDF and ADF presented quadratic behaviors, with a reduction of 12% in the concentration of NDF and 13% in the concentration of ADF compared to the control treatment.

**Table 3:** Chemical composition and carbohydrate fractionation (g.Kg^-1^) of corn silage (g.kg^-1^) treated with lignocellulolytic enzymatic complex.

Parameters	Enzimatic activity (U. min). kg^-1^ NM				SEM	P		
	0	10	20	30		Linear	Quadratic	RE
pH	3.63	3.71	3.71	3.60	0.10	0.20	0.22	-
Dry matter	316	317	305	305	0.33	<0.01	<0.01	*Y* = −0.04*x*^2^ − 0.034*x* + 31.69
Organic matter	972	970	967	963	0.09	0.10	0.21	-
Crude protein	64.0	63.2	67.4	67.5	0.08	0.56	0.10	-
ADIN	1.98	1.79	1.53	1.60	0.42	0.06	0.01	*Y* = 0.06*x*^2^ − 0.032*x* + 1.99
Ethereal Extract	24.4	21.1	21.4	21.2	0.05	0.88	0.37	-
Neutral detergent fiber (NDF)	497	473	434	439	0.98	<0.01	<0.01	*Y* = 0.074*x*^2^ − 0.43*x* + 39.93
Indigestible NDF	85.6	63.6	52.0	51.3	0.94	0.02	0.01	*Y* = 0.057*x*^2^ − 2.89*x* + 75.89
Acid detergent fibe	302	282	260	272	0.42	0.01	0.19	*Y* = −0.081*x*^2^ − 3.56*x* + 248.0
Total Carbohydrates	884	886	879	875	0.13	<0.01	0.09	*Y* = −0.034*x* + 88.59
Non-fibrous carbohydrates	387	414	445	435	1.09	<0.01	0.05	*Y* = −0.089*x*^2^ + 0.44*x* = 48.5
Cellulose	217	214	204	215	0.60	0.36	<0.01	*Y* = 0.08*x*^2^ − 0.082*x* + 22.63
Hemicellulose	195	191	174	167	1.26	<0.01	<0.01	*Y* = −1.0076*x* + 152.08
Lignin (ABSL)	85.2	67.5	56.1	57.1	3.83	<0.01	0.10	*Y* = −0.0957*x* + 8.08
Lignin (PPSL)	66.4	49.5	36.8	36.9	2.78	<0.01	0.10	*Y* = −0.1012*x* + 6.25
Fraction A + B1	438	467	507	497	0.98	<0.01	0.07	*Y* = 0.3523*x* + 54.38
Fraction B2 * ^1^	458	459	430	437	0.98	<0.02	0.06	*Y* = −0.2078*x* + 35.80
Fraction C * ^1^	115	86.4	67.3	69.0	1.12	<0.03	0.08	*Y* = −0.1468*x* + 8.51

SEM- Mean standard error;

* Fraction calculated on the basis of the lignin obtained by the analysis with acetyl bromide

^1^- values expressed as a function of total carbohydrates;

Lignin (ABSL): lignin determined by oxidation with acetyl bromide; Lignin (PPSL): lignin determined by oxidation with potassium permanganate; Acid lignin: lignin determined with 12M sulfuric acid; RE—Regression equation; IP—Inflection point

The concentration of hemicellulose in corn silage presented a linear decrease of 1.0 g.kg^-1^ for each mg of addition of the enzymatic complex. The cellulose content presented a quadratic behavior, with a 6% reduction compared to the control treatment. The lignin concentration showed a linear reduction of 0.10 g.kg^-1^ for each mg of the enzymatic complex added.

The fractionation of carbohydrates of corn silage presented a linear behavior in all fractions. For each mg of addition of the enzymatic complex, the digestible fractions (A + B1) showed a linear increase of 0.35 g.kg^-1^, the fraction with potential degradation (B2) presented a reduction of 0.20 g.kg^-1^, and the fraction considered totally indigestible (C) presented a reduction of 0.14 g.kg^-1^.

For sugarcane silage, a smaller amount of enzyme was required to produce beneficial effects similar to those observed for corn silage (Table 4). The addition of the enzymatic complex to the sugarcane silages altered the NFC content, which presented a quadratic behavior, with a 7% increase in the inflection point compared to the control treatment, according to the regression equations. The levels of NDF and ADF of sugarcane silage presented quadratic behaviors, with a reduction of 6% and 5%, compared to the control treatment, respectively. The concentration of hemicellulose had a linear reduction of 0.05 g.kg^-1^ for each mg of the enzymatic complex added. The cellulose content presented a quadratic behavior, with a 3% decrease compared to the control. There was a 57% decrease in ADIN compared to the control.

**Table 4:** Chemical composition and fractionation of carbohydrates of sugarcane silage (g.kg-1) treated with lignocellulolytic enzymatic complex.

Parameters	Enzimatic activity (U. min). kg^-1^ NM				SEM	P				
	0	10	20	30		Linear	Quadratic	RE	R^2^	IP
pH	3.45	3.45	3.48	3.48	0.01	0.25	0.41	-	-	-
Dry matter	269	259	255	260	0.33	0.24	0.23	-	-	-
Organic matter	972	968	967	964	0.07	0.08	0.09	-	-	-
Crude protein	34.6	38.7	39.0	39.9	0.06	0.05	0.12	*Y* = 0.01*x* + 3.56	*R*^*2*^ *= 0*.*78*	-
ADIN	0.72	0.54	0.31	0.37	0.03	0.02	0.03	*Y* = 0.006*x*^2^ − 0.03*x* + 0.74	*R*^*2*^ *= 0*.*94*	*18*.*7*
Ethereal Extract	17.2	16.9	16.9	16.6	0.49	0.22	0.23	-	-	-
Neutral detergent fiber (FDN)	669	623	629	630	0.31	0.01	0.01	*Y* = 0.01*x*^2^ − 0.46*x* + 49.44	*R*^*2*^ *= 0*.*88*	*10*.*9*
Indigestible NDF	143	134	133	134	0.53	0.03	0.01	*Y* = 0.02*x*^2^ − 1.08*x* + 142.9	*R*^*2*^ *= 0*.*96*	*10*.*6*
Fiber in acid detergent	412	382	390	389	0.13	0.01	0.19	*Y* = 0.072*x*^2^ − 0.27*x* + 32.9	*R*^*2*^ *= 0*.*79*	*10*.*7*
Total Carbohydrates	920	913	911	907	0.61	<0.01	<0.01	*Y* = −0.039*x* + 91.89	*R*^*2*^ *= 0*.*90*	-
Non-fibrous carbohydrates	251	290	283	277	0.3	<0.01	0.02	*Y* = −0.02*x*^2^ + 0.15*x* + 46.5	*R*^*2*^ *= 0*.*81*	*10*.*5*
Cellulose	302	286	294	293	0.28	0.03	0.06	*Y* = 0.06*x*^2^ − 0.26*x* + 31.84	*R*^*2*^ *= 0*.*77*	*10*.*3*
Hemicellulose	257	241	239	241	1.79	<0.01	<0.01	*Y* = −0.0505*x* + 16.0	*R*^*2*^ *= 0*.*57*	-
Lignin (ABSL)	110	96.7	96.1	96.3	1.64	<0.01	<0.01	*Y* = 0.03*x*^2^ − 0.14*x* + 10.94	*R*^*2*^ *= 0*.*94*	*15*.*4*
Lignin (PPSL)	91.2	78.7	76.7	76.1	1.14	0.01	0.01	*Y* = 0.03*x*^2^ − 0.136*x* + 9.07	*R*^*2*^ *= 0*.*97*	*23*.*2*
Fraction A + B1	320	373	374	368	0.93	0.01	0.02	*Y* = −0.01*x*^2^ − 0.48*x* + 45.8	*R*^*2*^ *= 0*.*91*	*11*.*3*
Fraction B2 * ^1^	529	486	488	494	0.8	0.02	0.02	*Y* = 0.012*x*^2^ − 0.46*x* + 52.7	*R*^*2*^ *= 0*.*92*	*11*.*9*
Fraction C * ^1^	151	141	137	139	0.1	0.08	0.02	*Y* = 0.069*x*^2^ − 0.29*x* + 13.2	*R*^*2*^ *= 0*.*95*	*12*.*7*

SEM- Mean standard error;

* Fraction calculated on the basis of the lignin obtained by the analysis with acetyl bromide;

^1^- values expressed as a function of total carbohydrates;

Lignin (ABSL): lignin determined by oxidation with acetyl bromide; Lignin (PPSL): lignin determined by oxidation with potassium permanganate; Acid lignin: lignin determined with 12M sulfuric acid; RE—Regression equation; IP—Inflection point.

The fractionation of carbohydrates from sugarcane silage showed a quadratic behavior, with the most digestible fractions (A + B1) increasing by 20%, the less digestible fraction with potential degradation (B2) decreased by 7%, and the fraction considered totally indigestible (C) decreased by 9%. The values in percentages were obtained from the inflection point compared to the control treatment. In the sugarcane silage, quadratic effects on the lignin concentration were observed, with a reduction of 12.5% compared to the control, which was reflected in the monomeric composition.

The monomeric composition of lignin from both silages was altered by the action of the enzymatic complex (Table 5). In corn silage, the *p*-hydroxyphenyl (H) monomer increased linearly by 0.51 g.kg^-1^, guaiacyl (G) increased by 1.44 g.kg^-1^, and syringyl increased by 1.63 g.kg^-1^ for each mg of enzymatic complex addition, according to the analysis of regression equations presented in Table 6. In the sugarcane silage, all the monomers presented quadratic behavior, with the inflection point occurring close to the concentration of 20 mg kg^-1^ NM. The *p*-hydroxyphenyl (H) monomer increased by 97%, guaiacyl (G) increased by 23%, and syringil increased 140% compared to the control treatment.

**Table 5:** Monomeric composition (ug / mg protein-free cell wall) of corn and sugarcane silages treated with increasing levels of lignocellulolytic enzymes.

Parameters	Enzimatic activity (U. min). kg^-1^ NM				SEM	P				
	0	10	20	30		Linear	Quadratic	RE	R^2^	IP
Corn Silage										
*p*-Hydroxyphenyl (H)	36.47	43.65	47.87	52.28	2.02	0.02	0.15	*Y* = 0.516*x* + 37.31	*R*^*2*^ *= 0*.*98*	-
Guaiacil (G)	107.0	151.8	153.0	154.6	6.93	0.01	0.21	*Y* = 1.44*x* + 120.02	*R*^*2*^ *= 0*.*64*	-
Syringe (S)	46.14	95.67	97.27	100.08	7.75	0.02	0.17	*Y* = 1.6341*x* + 60.28	*R*^*2*^ *= 0*.*66*	-
S / G	0.43	0.63	0.64	0.65	0.03	0.02	0.24	*Y* = 0.0065*x* + 0.48	*R*^*2*^ *= 0*.*66*	-
Sugarcane Silage										
*p*-Hydroxyphenyl (H)	74.22	126.59	146.75	83.53	3.46	0.01	0.02	*Y* = −0.289*x*^2^ + 9.14*x* + 71.66	*R*^*2*^ *= 0*.*96*	*17*.*8*
Guaiacil (G)	181.9	207.2	225.4	221.5	1.97	0.02	0.02	*Y* = −0.0731*x*^2^ + 3.56*x* + 181.2	*R*^*2*^ *= 0*.*99*	*19*.*7*
Syringe (S)	116.0	221.5	279.3	234.4	6.91	0.01	0.01	*Y* = −0.3759*x*^2^ + 15.40*x* + 113.2	*R*^*2*^ *= 0*.*98*	*18*.*9*
S / G	0.64	1.07	1.24	1.06	0.08	0.03	0.02	*Y* = −0.0015*x*^2^ + 0.060*x* + 0.633	*R*^*2*^ *= 0*.*99*	*19*.*5*

SEM—Mean standard error; RE—Regression equation; PI—Inflection point.

**Table 6:** Cumulative in vitro gas production of silages treated with lignocellulolytic enzymatic complex.

Parameters	Enzimatic activity (U. min). kg^-1^ NM				SEM	P		
	0	10	20	30		Linear	Quadratic	RE
Corn Silage								
A (mL/100 mg SDM)	5.58	9.92	9.51	8.50	0.32	0.64	0.01	*Y* = 0.0121*x*^2^ + 0.430*x* + 6.29
B (/hour)	0.11	0.19	0.25	0.25	0.01	0.00	0.11	*Y* = 0.0040*x* + 0.146
Lag (hour)	1.41	0.05	0.01	0.01	0.02	0.00	0.46	*Y* = −0.0278*x* + 0.588
D (mL/100 mg DM)	12.2	11.7	14.0	11.1	0.32	0.04	<0.01	*Y* = −0.0081*x*^2^ + 0.269*x* + 10.83
E (/hour)	0.03	0.04	0.04	0.04	0.01	0.27	0.01	*Y* = −0.00002*x*^2^ + 0.00103*x* + 0.02
Total (mL/100mg DM)	17.8	21.6	23.5	16.6	0.44	0.31	<0.01	*Y* = −0.0203*x*^2^ + 0.7006*x* + 17.12
Sugarcane Silage								
A (mL/100 mg SDM)	10.6	12.5	10.4	9.6	0.30	0.07	0.02	*Y* = −0.0075*x*^2^ + 0.184*x* + 10.71
B (/hour)	0.24	0.31	0.32	0.26	0.02	0.34	0.08	-
Lag (hour)	0.009	0.005	0.001	0.003	0.001	0.02	0.04	*Y* = −0.0095*x*^2^ − 0.0002*x* + 0.105
D (mL/100 mg DM)	7.32	10.49	8.28	6.80	0.32	0.19	< 0.01	*Y* = −0.012*x*^2^ + 0.374*x* + 7.51
E (/hour)	0.04	0.04	0.03	0.04	0.00	0.38	0.20	-
Total (mL/100mg DM)	17.9	22.9	18.7	16.4	0.57	0.09	<0.01	*Y* = −0.020*x*^2^ + 0.53*x* + 18.22

EPM: Mean standard error; A: gas volume of the fast degradation fractions (soluble sugars and starch); B: rate of degradation per hour of the fast fraction; Lag: time of colonization and beginning of fermentation in hours; D: gas volume of slow degradation fractions (cellulose and hemicellulose); E: rate of degradation per hour of the slow fraction; Total: cumulative gas production in vitro (mL / 100 mg MS incubated); RE—Regression equation; PI—Inflection point.

Table 6 shows the results regarding the *in vitro* gas production by the corn and sugarcane silages. For fractions of rapid degradation of corn silage, increases of 70% in gas production were observed compared to the control. The degradation rate presented a linear increase. The time of colonization and initiation of *in vitro* fermentation (*lag time*) in corn silage was linearly reduced by 0.02 hours/percentage unit of the enzymatic complex. The fractions of slow ruminal degradation in corn silage presented increases of 14% in gas production and 36% in the degradation rate, respectively, compared to the control. The total cumulative gas production in corn silage had a 32% increase compared to the control treatment.

In the sugarcane silage, there was a quadratic effect on in vitro gas production for the fraction of rapid degradation, with an increase of 18%. The time of colonization and initiation of *in vitro* fermentation decreased by 89%. For the slow degradation fractions, there was a quadratic behavior, with a 43% increase in the gas production, and there was no effect of the addition of the enzymes on the rate of degradation of these fractions. The cumulative production of gases in sugarcane silage increased by 28% compared to the control.

Table 7 shows the concentrations of polyphenols and flavonoids and the antioxidant capacity of corn and sugarcane silages, respectively. These parameters were influenced in an increasing linear manner with the addition of the enzymatic complex at concentrations of 0 to 30 mg kg^-1^. In corn silage, there was a linear increase of 0.68 mg.kg^-1^ of polyphenols, 0.06 mg.kg^-1^ of flavonoids, 3.76 μM.kg^-1^ of antioxidant capacity, and 0.09 mg.kg^-1^ of reducing power for each mg of inclusion of the enzymatic complex. In sugarcane silage, there was a linear increase of 3.97 mg.kg^-1^ of polyphenols, 0.42 mg.kg^-1^ of flavonoids, 18.18 μM.kg^-1^ of antioxidant capacity, and 0.47 mg.kg^-1^ of reducing power for each mg of inclusion of the enzymatic complex.

**Table 7:** Polyphenols, flavonoids, antioxidant capacity and reducing power of silages treated with lignocellulolytic enzymatic complex.

Parameters	Enzimatic activity (U. min). kg^-1^ NM				SEM	P		
	0	10	20	30		Linear	Quadratic	RE
Corn Silage								
Polyphenols (mg GAE / 100g)	77.91	79.92	91.49	96.96	3.66	0.01	0.15	*Y* = 0.684*x* + 76.26
Flavonoids (mg QE / 100g)	9.04	9.71	10.03	11.17	0.35	<0.01	0.12	*Y* = 0.066*x* + 8.98
Antioxidant capacity (uM))	135.0	153.0	211.3	241.1	19.81	0.02	0.21	*Y* = 3.762*x* + 128.64
Reducing power (mg GAE/ 100g)	13.38	13.68	14.25	16.43	0.65	0.03	0.17	*Y* = 0.097*x* + 12.98
Sugarcane Silage								
Polyphenols (mg / 100g)	412.83	474.71	501.34	536.37	20.87	0.01	0.16	*Y* = 3.972*x* + 421.72
Flavonoids (mg / 100g)	34.70	35.20	40.90	46.80	2.62	0.01	0.27	*Y* = 0.42*x* + 33.1
Antioxidant capacity (uM))	2961.6	3042.2	3401.7	3448.0	98.85	<0.01	0.19	*Y* = 18.18*x* + 2940.6
Reducing power (mg / 100g)	55.90	60.70	65.30	70.10	2.44	<0.01	0.14	*Y* = 0.472*x* + 55.92

SEM—Mean standard error; RE—Regression equation.

## Discussion

Before starting the discussion, it is important to mention that enzymes that act on lignin act in an aerobic environment. Therefore, the anaerobic conditions present in the ensiling process would not favor the action of these enzymes. However, tests were performed to determine the time of action of the enzyme complex on the cell wall (Table 8) and it was observed that the action of this enzyme complex is rapid (less than 30 minutes). Thus, it is likely that the action of enzymes on lignin occurred before vacuuming the bags.The activity of the lignocellulosic enzymes added to corn silage decreased the concentrations of the main components of the cell wall, but increased the content of non-fibrous carbohydrates, as can be observed in the recovery of carbohydrate fractions, shown in Table 2. These data demonstrate that in the fermentative process of ensiling, the microorganisms consume part of the carbohydrates present in the cellular wall. The addition of the enzymatic complex accentuated this effect mainly on the lignin fraction, which resulted in a higher amount of non-fibrous carbohydrates in both silages, which might reflect a better use of forage by ruminants. It is noteworthy that the reduction in lignin recovery was higher for sugarcane than corn silage, probably due to its higher concentration in this forage.

**Table 8:** Neutral detergent fiber and acid detergent fiber content of corn silage and coast cross hay treated with 20 mg.kg-1 MN of a compound of lignocellulolytic enzymes produced by white rot fungus for 30, 60 and 120 minutes.

	Without enzyme	With enzyme				
		Time (minutes)				
		30	60	120	SEM	P
Parameters	Corn silage				-	-
Neutral detergent fiber (g.kg^-1^)	495a	432.9b	433.3b	433b	0.39	0.002
Acid Detergent Fiber (g.kg^-1^)	303.3a	261.7b	260.4b	260.6b	0.36	< 0.001
	Sugar cane silage					
Neutral detergent fiber (g.kg^-1^)	668.0a	630b	630.5b	629.8b	0.65	< 0.001
Acid Detergent Fiber (g.kg^-1^)	415a	381.6b	381.4b	380.9b	0.69	0.003
	Hay coast cross					
Neutral detergent fiber (g.kg^-1^)	717.5a	705.7b	705.9b	706b	0.59	0.004
Acid Detergent Fiber (g.kg^-1^)	471.2a	447.9b	447.3b	448b	0.48	0.002

Averages followed by the same letter do not differ statistically from each other by the 5% tukey test; SEM—Mean standard error

The changes reflected in the chemical composition of the silages, and the variation in the extent of the reduction for the different cell wall fractions should be related to the composition of the enzymatic complex (Table 1) and to the stability of the enzymes, which act in broad ranges of pH and temperature.

In the process of cell wall digestion by the microorganisms present in the rumen, lignin is considered a limiting factor due to its close relationship with cellulose and, especially, with hemicellulose, which creates a physical barrier, making it difficult degrade these fractions by microorganisms [29]. Of the changes observed in the components of the cell wall, proportionally, lignin was the fraction that experienced the greatest effect of adding the enzymatic complex, both in corn silage and in sugarcane silage. This is probably due to the characteristics of the enzymes that act on lignin, which, although they have an optimal pH to maximize their activity, continue to act even in more acidic media, such as the inside of the silo. The decrease of lignin by 34% in corn silage was linear. In the sugarcane silage there was a reduction of 12.5%, and the effect was quadratic.

The decrease in the hemicellulose fraction in both silages is related to the concentration of the enzymes that make up the enzymatic complex; 54% of these enzymes act on xylan and mannan (xylanase and mannanase), which are the main compounds of hemicellulose. Another reason is that hemicellulose has branched chains, which are easier to break by the action of the enzymes. In addition, hemicellulose is also more closely linked to lignin [30], therefore, the decrease in lignin concentration favored the hydrolysis of hemicellulose.

The addition of the enzymatic complex decreased the cellulose concentration of the silages, but by a smaller proportion, compared to the other fractions of the cell wall, probably due to the more acidic pH found in the silo. carboxymethylcellulase represented 33% of the enzymatic complex used. The cellulose bundles are dispersed in a matrix of hemicellulose and lignin, and for the enzyme to gain access to this fraction, the matrix must be broken first. Therefore, when the carboxymethylcellulase of the enzymatic complex had access to the cellulose, the medium was likely already acidifying in the silo, and thus the enzymatic activity would have been diminished. The highest activity of carboxymethylcellulase under the experimental conditions was observed at pH 6.0 (data no show). In addition, the cellulose chain is more linear and, therefore, more difficult to break than hemicellulose, for example.

The monomeric composition of lignin was also modified in both silages by the addition of the enzymatic complex. The guaiacyl (G) monomer was present in the highest amount, followed by syringyl (S) and *p*-hydroxyphenyl (H). However, the S monomer had a higher increase, compared to the other monomers, which made the S/G ratio larger. This increase in the S/G ratio suggests that the lignin composition after the enzymatic treatment was less condensed [31], which probably facilitated the entry of the microorganisms to access the nutritional components.

The decrease in the carbohydrate fractions that compose the cell wall was reflected in the increase in the non-fibrous carbohydrates, modifying the composition of the carbohydrates present in the silages. The A + B1 fraction, which corresponds to sugars and starch, increased in both silages. It is likely that the increase in these fractions favors the production of short chain fatty acids (CFA), especially propionic acid, which is the main precursor of glucose for ruminants [32].

The decrease in ADIN, which represents the fraction of nitrogen bound to lignin and which is unavailable to the microorganisms in the rumen and to the animal, in corn silage was 22% and in sugarcane silage was 57%. This suggests that the action of the enzymatic complex on lignin, in addition to the changes in the chemical composition of the cell wall of the silages, can reduce the excretion of unavailable nitrogen and increase N retention by the animal.

The 32% increase in cumulative total gas production *in vitro* for corn silage at the inflection point compared to the control treatment is caused mainly by increases in the cumulative gas production of the fraction of rapid degradation. The rate of degradation of this fraction, together with the reduction in colonization time, might have produced the increase in propionate production. These gas production data are supported by the chemical composition data, which showed that the NDF and lignin contents were reduced and the NFC and A + B1 contents of the carbohydrates were increased by the addition of enzymes. The observed values are of the same magnitude.

However, for sugarcane silage, although there was a significant increase in the cumulative total gas production of 28% and in the colonization time, the contribution of gas production from the slow degradation fraction was higher than of the fast degradation fraction. The lower cumulative gas production in sugarcane silage than corn silage is due to the lower fiber quality in sugarcane that has slow ruminal degradation [33]. For the slow degradation fraction, the increase was 42%, and for the rapid degradation fraction, the increase was 18% in their respective points of inflection.

The colonization time decreased by 99% in corn silage and by 88% in sugarcane silage with the addition of the lignocellulolytic enzymatic complex. This parameter is related to the rumen degradation of feed and is probably responsible for the increase in degradation rates. In addition, a decrease in colonization time might increase the ruminal passage rate, which is one of the most important parameters that influences feed intake [34].

In accordance with the results obtained in this study, increased forage digestibility has been observed when a commercial fibrolytic enzyme mixture was used in corn silage [35]. In addition, the use of sugarcane inoculated with *Pleurotus sapidus* resulted in increased total gas production, and improved digestibility and fermentative parameters of silage [36]. Furthermore, Cysneiros et al. [37] observed that the addition of fibrolytic enzyme levels (0, 5, 10, and 20 mg of enzymes per kg of natural matter) to the chemical composition of corn silage did not alter the DM content of the silage. However, there was an increase in the crude protein content from 8.77% (control treatment) to 10.14% at the level of 20 mg and reduction in the NDF content from 49.96% (control treatment) to 47.25% at the 10 mg level.

In relation to the antioxidant profile, the addition of the enzymatic complex to both silages increased the concentration of polyphenols and flavonoids. This increase can be indicative of the monomers from the degradation of the lignin, since the monomers have a phenol group in their structure, making them responsive to the Folin-Ciocalteau reagents and aluminum chloride commonly used in the quantification of these molecules.

The antioxidant capacity, indicating the presence of compounds capable of reducing the ABTS • radical in the sample, and possibly the presence of lignin monomers, had a linear increase of 3.76 μM and 18.18 μM for each mg increase in inclusion of the enzymatic complex in corn and sugarcane silages, respectively. Although the absolute values were higher in sugarcane silage, the degradation of lignin increased the antioxidant capacity by a larger amount in the corn silage. At the concentration of 20 mg kg^-1^ NM, the corn silage increased its antioxidant capacity by 56% vs. a 14% increase in sugarcane silage compared to the control treatment.

The reducing power presented linear increases of 0.09 mg and 0.47 mg for each mg increase in the inclusion of the enzymatic complex in the corn and sugarcane silages, respectively. This analysis reflects the ability to reduce ferric ions, and is related to the amount of phenolic compounds in a sample [38]. Thus, these results confirm the action of the enzymatic complex on the release of lignin monomers.

## Conclusion

The addition of the lignocellulolytic enzymatic complex has an effect on the cell wall of corn and sugarcane silages, and produces decreases in the lignin concentration and particle colonization time, and increases in *in vitro* gas production.

There is also an increase in the antioxidant capacity and phenolic compound concentration of both corn silage and sugarcane silage with the addition of the enzymatic complex.

In corn silage, the best responses are observed at the concentration of 20 mg kg^-1^ NM of enzymatic complex. In sugarcane silage, the results are best at the concentration of 10 mg kg^-1^ NM of enzymatic complex.

Acknowledgment to the Coordination of Improvement of Higher Education Personnel (CAPES) for the scholarships granted, which allowed the conduction of the experiments and National Council for Scientific and Technological Development (CNPq) for project financing.

## Supporting information

S1 File Minimal data set. (XLSX)

S2 File ARRIVE Guidelines Checklist. (DOCX)
