# Supplementary material for: Reduction in lignin content and increase in the antioxidant capacity of corn and sugarcane silages treated with an enzymatic complex produced by white rot fungus
Source: PLoS One. 2020 Feb 21;15(2):e0229141. doi: 10.1371/journal.pone.0229141 (PMC7034799; doi:10.1371/journal.pone.0229141)

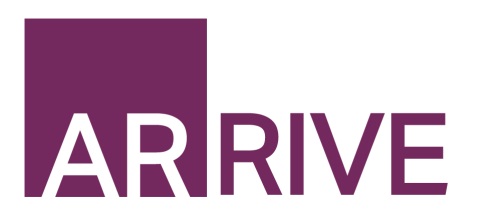


The ARRIVE Guidelines Checklist

Animal Research: Reporting In Vivo Experiments

Carol Kilkenny^1^, William J Browne^2^, Innes C Cuthill^3^, Michael Emerson^4^ and Douglas G Altman^5^

*^1^The National Centre for the Replacement, Refinement and Reduction of Animals in Research, London, UK, ^2^School of Veterinary Science, University of Bristol, Bristol, UK, ^3^School of Biological Sciences, University of Bristol, Bristol, UK, ^4^National Heart and Lung Institute, Imperial College London, UK, ^5^Centre for Statistics in Medicine, University of Oxford, Oxford, UK.*

|  | | ITEM | RECOMMENDATION | Section/ Paragraph |
| --- | --- | --- | --- | --- |
| 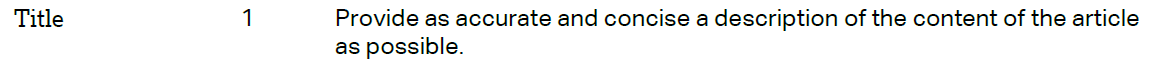 | | | Title |  |
| 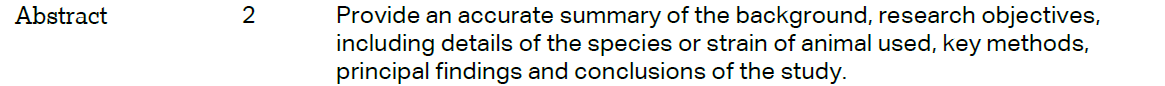 | | | Abstract |  |
| INTRODUCTION | | |  |  |
| 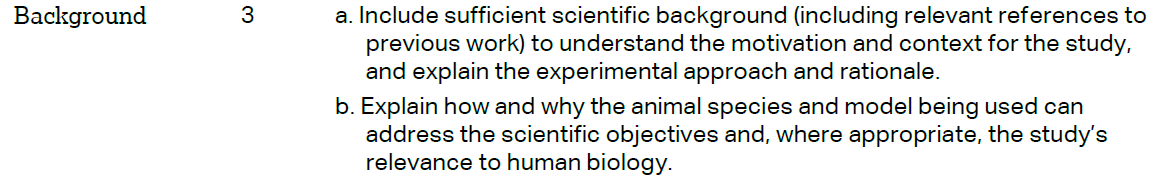 | | | Paragraphs  1-4  Paragraph 1 |  |
| 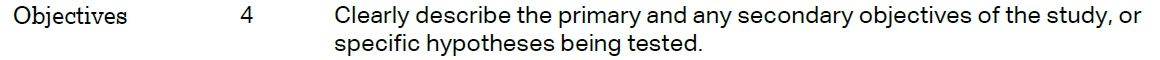 | | | Paragraph 5 |  |
| METHODS | | |  |  |
| 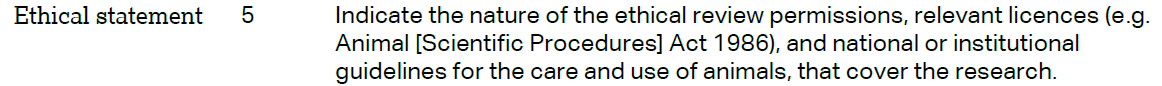 | | | Paragraph 1 |  |
| 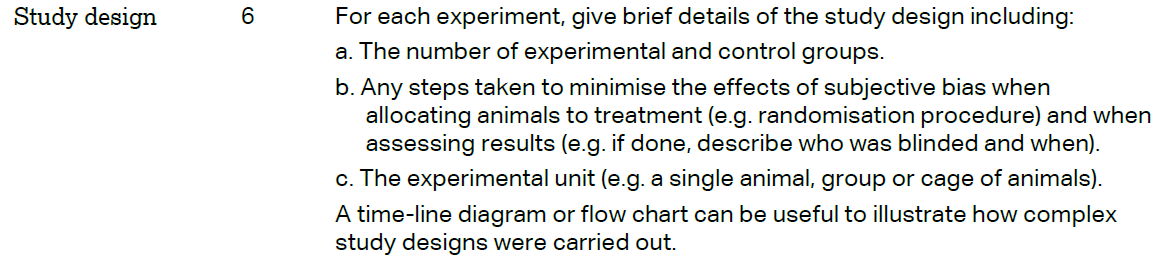 | | | Paragraph 5 |  |
| 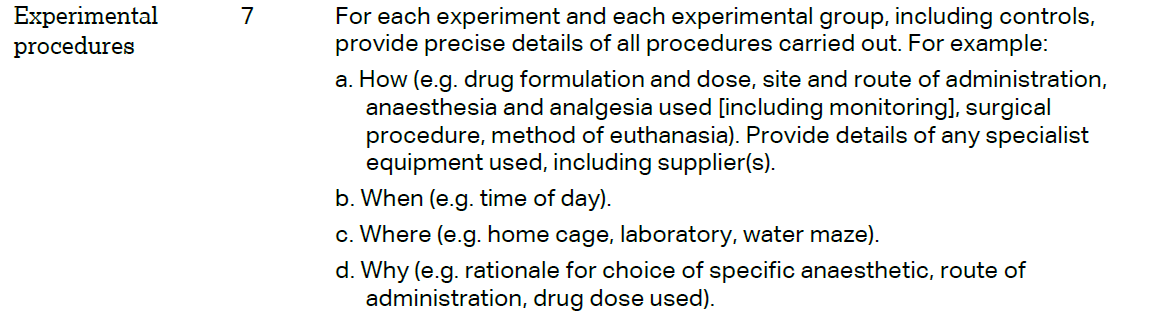 | | | not applicable |  |
| 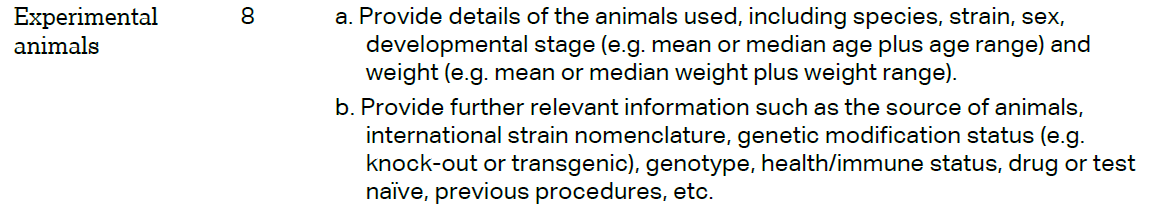 | | | Paragraph 20 |  |

The ARRIVE guidelines. Originally published in *PLoS Biology*, June 2010^1^

| 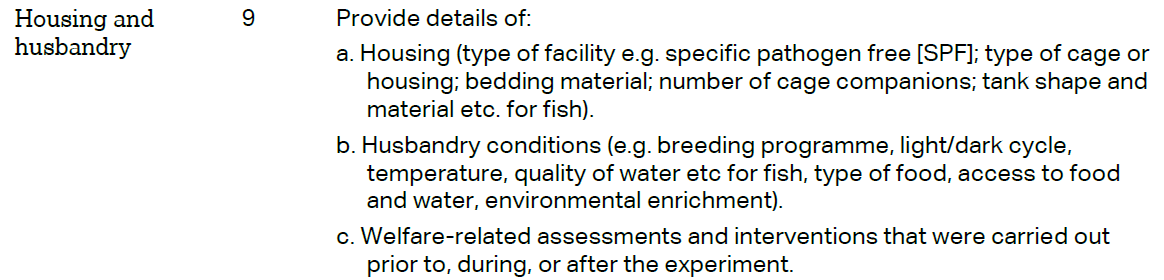 | not applicable | |
| --- | --- | --- |
| 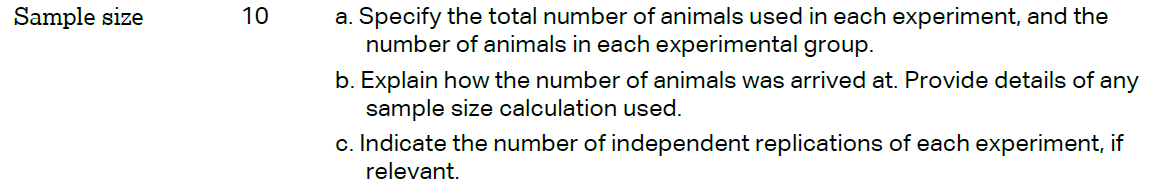 | Paragraph 20 | |
| 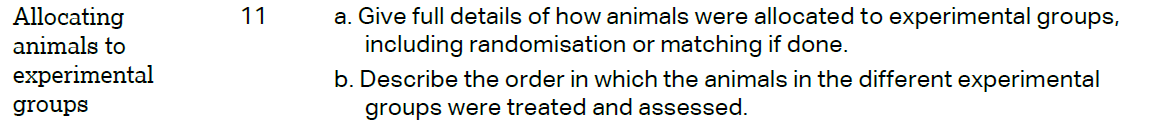 | Paragraph 20 | |
| 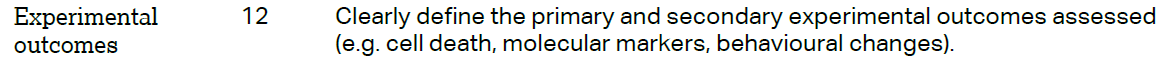 | not applicable | |
| 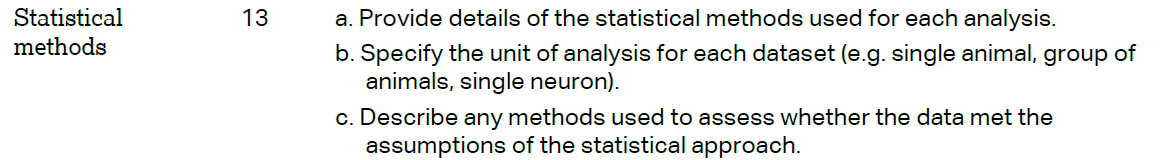 | not applicable | |
| RESULTS |  | |
| 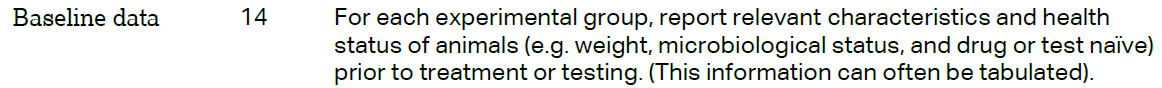 | not applicable | |
| 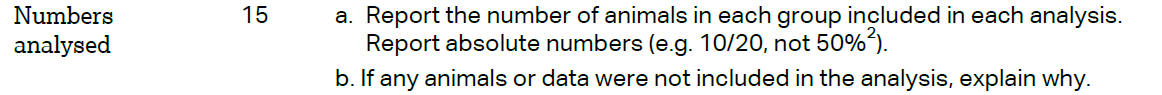 | Paragraph 8 | |
| 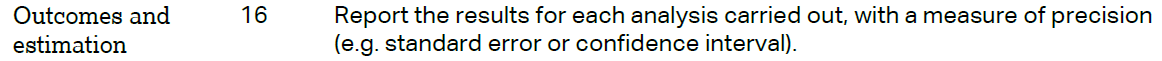 | Paragraph 8 | |
| 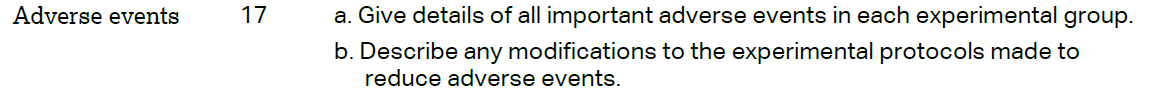 | not applicable | |
| DISCUSSION |  | |
| 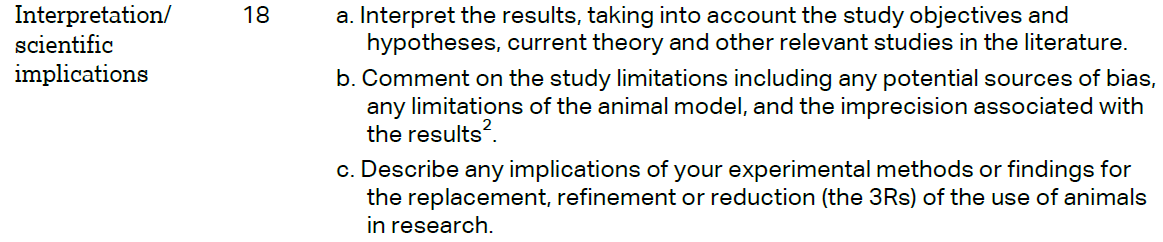 | Paragraphs 2-16 | |
| 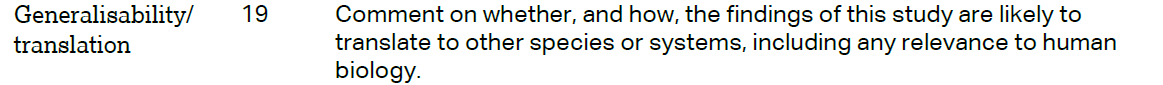 | not applicable | |
| 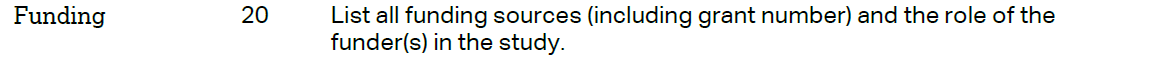 | | Paragraph 20 |


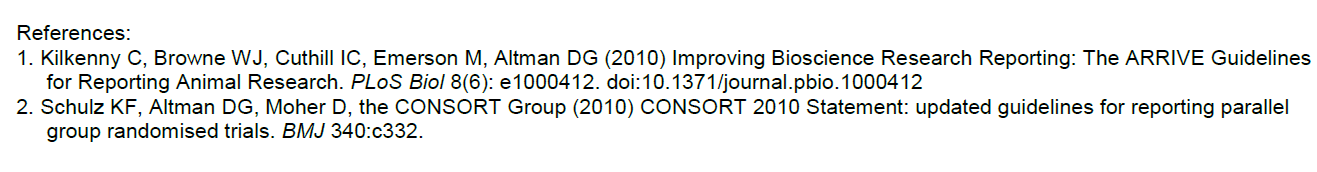

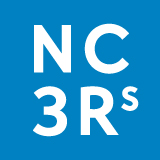

Supplement: S2 File — (DOCX) [file pone.0229141.s002.docx]
